# Pediatric Focal Nodular Hyperplasia Mimicking Hepatic Metastasis from Wilms Tumor: A Case Report

**DOI:** 10.70352/scrj.cr.26-0094

**Published:** 2026-04-29

**Authors:** Wataru Miyoshino, Katsuhiro Ogawa, Takashi Masuda, Shun Nakamura, Yuiko Nagasawa, Yushi Kaisyakuji, Hiroomi Takayama, Yoko Kawano, Teijiro Hirashita, Yuichi Endo, Masafumi Inomata

**Affiliations:** 1 Department of Gastroenterological and Pediatric Surgery, Oita University Hospital, Yufu, Oita, Japan; 2 Department of Comprehensive Surgery for Community Medicine, Oita University Faculty of Medicine, Yufu, Oita, Japan

**Keywords:** focal nodular hyperplasia, liver metastasis, Wilms tumor, indocyanine green (ICG) fluorescence

## Abstract

**INTRODUCTION:**

Focal nodular hyperplasia (FNH) is a rare benign liver lesion in children, representing approximately 2% of pediatric liver tumors and occurring far less frequently than in adults. Preoperative diagnosis is often challenging because FNH can closely mimic malignant or metastatic liver tumors on imaging, particularly in patients with a history of malignancy. Indocyanine green (ICG) fluorescence imaging has emerged as a useful intraoperative navigation tool in pediatric liver surgery; however, its role in distinguishing FNH from metastatic lesions remains underexplored.

**CASE PRESENTATION:**

A 5-year-old girl with a history of stage IV Wilms tumor—treated with systemic chemotherapy and in complete remission since 3 years of age—underwent routine follow-up imaging. MRI identified a small hepatic lesion in segment 1 (S1) that gradually enlarged on serial examinations. Contrast-enhanced CT demonstrated a 10-mm nodule with arterial phase enhancement and gradual relative washout in the portal and venous phases, raising concern for hepatic metastasis. Given the oncologic history and indeterminate imaging findings, laparoscopic partial hepatectomy of S1 was performed for diagnostic confirmation and treatment. For intraoperative localization, ICG (0.5 mg/kg body weight) was administered intravenously 36 h preoperatively. The lesion was not detectable under conventional white-light laparoscopy; however, near-infrared fluorescence imaging revealed diffuse fluorescence corresponding to the tumor location, enabling precise resection with minimal loss of hepatic parenchyma. Histopathological examination confirmed FNH, characterized by hyperplastic hepatocytes with proliferating bile ductules and small vessels, and a central stellate scar. The postoperative course was uneventful, with no evidence of recurrence at 9-month follow-up.

**CONCLUSIONS:**

Differentiating pediatric FNH from metastatic liver tumors remains challenging. Intraoperative ICG fluorescence imaging may assist not only with tumor localization but also by providing supplementary information about tumor characteristics, potentially facilitating safe and minimal hepatic resection. In this case, the fluorescence pattern was suggestive of FNH in retrospect. Further case accumulation is needed to clarify the utility of ICG fluorescence patterns for differential diagnosis and surgical planning in pediatric liver lesions.

## Abbreviations

FNH
: focal nodular hyperplasia
Gd-EOB-DTPA
: gadolinium-ethoxybenzyl-diethylenetriamine pentaacetic acid
HCC
: hepatocellular carcinoma
HE
: hematoxylin and eosin
ICG
: indocyanine green
S1
: segment 1

## INTRODUCTION

Most hepatic tumors in children are malignant, with hepatoblastoma being the most common.^1)^ FNH is a rare benign liver lesion, representing approximately 2% of pediatric liver tumors,^2)^ with a lower incidence than in adults. FNH is frequently identified in pediatric patients with underlying conditions, including those who have undergone treatment for childhood malignancies or those with congenital biliary atresia.^2,3)^ Differentiating FNH from malignant or metastatic liver tumors can be difficult.^4–6)^ Moreover, pediatric FNH often lacks the typical radiologic features observed in adults, making preoperative diagnosis challenging and necessitating careful consideration of malignancy.

ICG fluorescence imaging has recently emerged as a valuable intraoperative navigation tool, enabling real-time tumor localization and more precise determination of resection margins. This technique is increasingly used in pediatric liver surgery, particularly for hepatoblastoma.^7–9)^ However, despite its growing application in pediatric hepatic malignancies, its potential utility in differentiating benign lesions such as FNH from metastatic lesions remains insufficiently explored.

Here, we report a pediatric case of FNH initially suspected to represent hepatic metastasis from Wilms tumor based on clinical context and imaging findings. This case highlights the potential role of ICG fluorescence imaging not only for intraoperative tumor localization but also as a complementary tool that may help anticipate tumor characteristics and support safe, minimal resection of small or atypical pediatric hepatic lesions when preoperative diagnosis is uncertain.

## CASE PRESENTATION

**Fig. 1:**
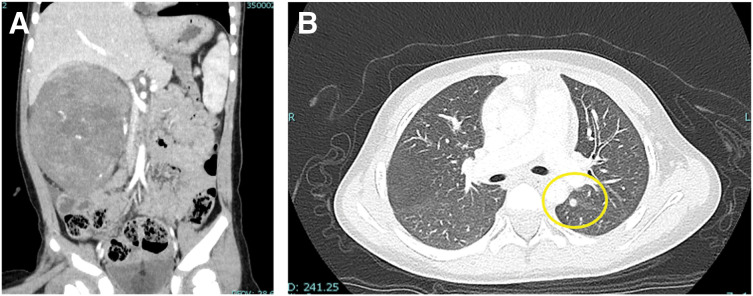
Initial contrast-enhanced CT findings at presentation. (**A**) Abdominal CT showing a 15-cm mass in the right kidney. (**B**) Chest CT demonstrating a 10-mm pulmonary metastasis in the left lung field (yellow circle).

A 2-year-old girl was referred to our institution for evaluation of a right-sided abdominal mass. Right nephrectomy was performed, and histopathological examination confirmed stage IV Wilms tumor with pulmonary metastases, ureteral invasion, and peritoneal dissemination (**Fig. 1A** and **1B**). Following the Children’s Oncology Group protocol, chemotherapy was initiated using the DD4A regimen (vincristine, dactinomycin, and doxorubicin). After 6 weeks of treatment, pulmonary and peritoneal metastases persisted, and therapy was escalated to regimen M (vincristine, doxorubicin, cyclophosphamide, carboplatin, and etoposide). Complete remission was achieved at 3 years of age.

**Fig. 2:**
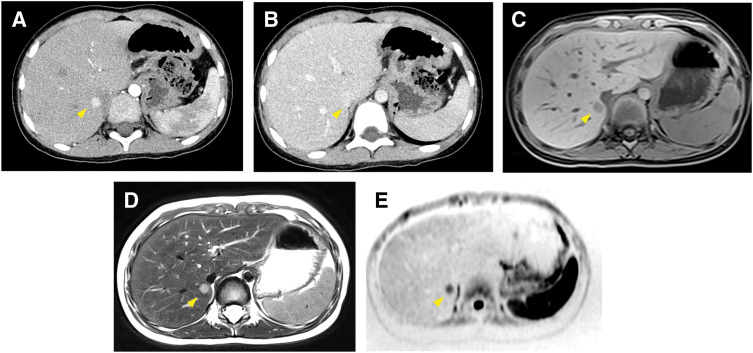
Imaging findings of the hepatic lesion. (**A**) Contrast-enhanced abdominal CT (arterial phase) showing a 10-mm hypervascular nodule in S1 (arrowhead). (**B**) Equilibrium phase demonstrating the nodule with contrast retention isodense to normal liver parenchyma (arrowhead). (**C**) T1-weighted MRI showing a hypointense nodule in S1 (arrowhead). (**D**) T2-weighted MRI demonstrating a hyperintense nodule in S1 (arrowhead). (**E**) Diffusion-weighted imaging revealing restricted diffusion within the lesion (arrowhead). S1, segment 1

At 5 years of age, routine follow-up MRI revealed a small hepatic lesion in S1, which gradually enlarged over time. Contrast-enhanced abdominal CT showed a 10-mm lesion in S1 with arterial phase enhancement followed by gradual relative washout in the portal and venous phases (**Fig. 2A** and **2B**). On MRI, the lesion was hypointense on T1-weighted imaging, hyperintense on T2-weighted imaging, and showed restricted diffusion (**Fig. 2C**–**2E**). A central scar—a characteristic feature of FNH—was not clearly identified. These imaging findings, together with lesion enlargement and the patient’s history of stage IV Wilms tumor, raised a strong suspicion of hepatic metastatic recurrence. Percutaneous biopsy was considered technically challenging because the tumor measured only 1 cm and was located in close proximity to the inferior vena cava. Although benign lesions such as FNH could not be fully excluded, the clinical context and imaging characteristics favored a diagnosis of metastasis. Therefore, laparoscopic partial hepatectomy of S1 was performed to obtain a definitive histological diagnosis and achieve complete resection.

**Fig. 3:**
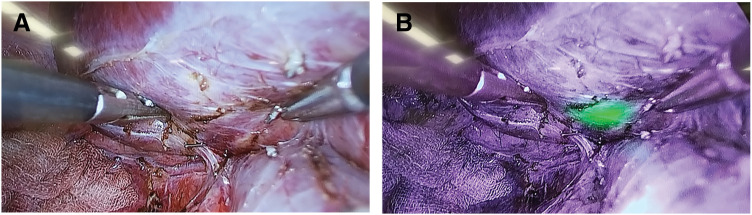
Intraoperative ICG imaging. (**A**) White-light laparoscopic examination showing no visible tumor on the liver surface. (**B**) Near-infrared fluorescence imaging clearly revealing fluorescence corresponding to the tumor location. ICG, indocyanine green fluorescence

**Fig. 4:**
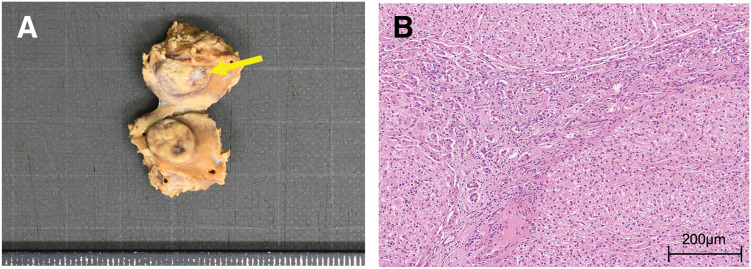
Macroscopic and histopathological findings. (**A**) Resected specimen (10 × 8 mm) showing a yellowish-white tumor with a central white scar (yellow arrow). (**B**) Histopathological examination showing hyperplastic hepatocytes with proliferating bile ductules, dilated capillaries, and collagenous fibers within the central scar (HE staining, ×100). HE, hematoxylin and eosin

ICG fluorescence imaging was planned to assist with intraoperative tumor localization because a definitive preoperative diagnosis could not be established and alternative tumor types remained possible. ICG was administered intravenously 36 h preoperatively at a dose of 11 mg (0.5 mg/kg body weight). During laparoscopic mobilization of the right hepatic lobe, the lesion was not visible under conventional white light; however, near-infrared fluorescence imaging clearly delineated fluorescence corresponding to the tumor location (**Fig. 3A** and **3B**). Fluorescence guidance enabled the precise laparoscopic resection of this small lesion that was otherwise undetectable using white light alone. The resected specimen measured 10 × 8 mm and contained a well-demarcated tumor with a central stellate scar visible on the cut surface (**Fig. 4A**). Histopathological examination demonstrated hyperplastic hepatocytes with minimal cytological atypia, along with proliferating small vessels and bile ductules, and collagenous fibers forming a central scar (**Fig. 4B**). These findings were consistent with the diagnosis of FNH. Immunohistochemical staining, including β-catenin, was not performed because the diagnosis was considered definitive based on the characteristic histopathological features. The postoperative course was uneventful, and no evidence of recurrence was observed during 9 months of follow-up.

## DISCUSSION

Pediatric FNH is a rare benign liver lesion and is often detected in patients with underlying conditions such as congenital biliary atresia or following pediatric cancer treatment.^2,3)^ Although FNH is benign, surgical resection may be warranted in symptomatic cases (e.g., abdominal pain) or when imaging findings are atypical, and malignancy cannot be excluded.^4)^ Diagnosis relies primarily on CT and MRI, with a central stellate scar considered a characteristic feature,^1,10)^ and optimally visualized on Gd-EOB-DTPA–enhanced MRI.^11)^ However, in children—especially those with prior cancer treatment or comorbidities—FNH may present as multiple small lesions and may lack typical imaging features, complicating differentiation from hepatoblastoma or metastatic liver tumors.^2,3)^ In the present case, the lesion was solitary and small and lacked a clearly identifiable central scar on imaging. Although Gd-EOB-DTPA–enhanced MRI may improve central scar visualization, a definitive diagnosis was considered unachievable by imaging alone in this small lesion, as percutaneous biopsy was technically unfeasible. In combination with the patient’s oncologic history, these factors favored suspected hepatic metastatic recurrence rather than FNH. Ultimately, resection was pursued to establish a definitive diagnosis.

ICG fluorescence imaging has gained significant attention as an intraoperative navigation tool for liver tumors. It is widely used in adult HCC and metastatic liver tumors, and it is also applied in pediatric tumors such as hepatoblastoma.^9,12–14)^ ICG is absorbed by hepatocytes and excreted into bile. Differences in vascular dynamics and biliary excretion between normal liver tissue and tumors can yield distinct fluorescence patterns that support tumor localization.^7)^ In this case, ICG (0.5 mg/kg) was administered intravenously 36 h prior to surgery. Although no universally standardized dosing protocol currently exists for pediatric patients, doses ranging from 0.1 to 0.5 mg/kg administered 24–90 h before surgery have been reported across published pediatric series.^15)^ ICG imaging was selected to facilitate intraoperative identification despite diagnostic uncertainty. Under near-infrared light, the lesion exhibited fluorescence at the tumor site, whereas it remained undetectable under standard white light.

Several studies have described ICG fluorescence-guided liver resection for adult FNH. Li et al.^16)^ reported the safety and efficacy of fluorescence-guided robotic liver resection in patients preoperatively diagnosed with FNH. In addition, FNH has been incidentally reported in studies involving HCC or metastatic tumors.^17,18)^ These reports describe diffuse fluorescence in FNH with sharp contrast against surrounding non-tumorous tissue, resembling the pattern reported in HCC and hepatoblastoma. In HCC, tumors are predominantly supplied by arterial blood flow; ICG may be absorbed but not effectively excreted by tumor cells, resulting in persistent fluorescence.^7)^ Hepatoblastoma is similarly known to exhibit ICG fluorescence through mechanisms analogous to those in HCC.^14)^ In contrast, ICG retention in FNH is generally attributed to impaired biliary excretion related to abnormal bile ducts. Histologically, malformed or ectatic bile ductules within fibrous septa may limit effective ICG excretion and produce diffuse fluorescence.^16)^ In the present case, bile ductular proliferation was observed within fibrous septa, and bile ducts required for ICG excretion were described as deficient, which may explain the observed fluorescence pattern. Although the ICG fluorescence patterns of HCC and hepatoblastoma can resemble that of FNH, an important distinction exists: the fluorescence pattern in HCC and hepatoblastoma varies considerably depending on tumor differentiation, degree of necrosis, and response to prior chemotherapy.^7,14)^ In contrast, FNH, owing to its benign and histologically uniform composition, demonstrates diffuse ICG staining in virtually all cases, as reported by Li et al.^16)^ In the present case, the uniform diffuse fluorescence pattern, combined with the patient's clinical profile—including age beyond the typical peak incidence of hepatoblastoma^1)^ and the absence of predisposing genetic conditions or underlying liver disease associated with HCC^1)^—made hepatoblastoma and HCC clinically unlikely and was retrospectively consistent with a diagnosis of FNH.

Experience with ICG fluorescence imaging in pediatric liver tumors remains limited, with most studies focusing on hepatoblastoma. Abdelhafeez et al.^9)^ reviewed ICG use in pediatric tumors and reported cases involving Wilms tumor pulmonary metastases, although ICG accumulation was not observed in those lesions. In pediatric metastatic liver tumors, neuroblastoma and rhabdomyosarcoma have been reported to exhibit ICG fluorescence^9)^; however, fluorescence patterns may vary according to tumor characteristics.^9)^ In our case, ICG was used for localization irrespective of the final diagnosis and enabled identification of a very small lesion. While intraoperative ICG fluorescence alone cannot confirm FNH, the fluorescence pattern observed, even in small lesions, may provide supplementary information that helps distinguish FNH from other pediatric metastatic liver tumors and supports the determination of an optimal resection margin. Important limitations should be noted: ICG imaging cannot detect lesions deeper than 1 cm from the liver surface^7,14)^ and cannot replace preoperative imaging or histopathological examination for definitive diagnosis.

## CONCLUSIONS

Differentiating pediatric FNH from metastatic liver tumors can be challenging. This case demonstrates that intraoperative ICG fluorescence imaging may serve not only for tumor localization but also for providing supplementary information regarding tumor characteristics. Although ICG fluorescence alone cannot provide a definitive diagnosis, the fluorescence pattern observed was suggestive of FNH in retrospect. This dual utility may facilitate optimal and minimal hepatic resection in pediatric patients with prior malignancy. Further case accumulation is needed to validate the utility of ICG fluorescence patterns for differential diagnosis and surgical planning.
